# Ammonia-Cyanuric Acid Co-Production in Boiler Denitrification System

**DOI:** 10.3390/ma17194692

**Published:** 2024-09-24

**Authors:** Qingjia Wang, Haowen Wu, Man Zhang, Qiang Song, Nan Hu, Hairui Yang

**Affiliations:** 1 School of Energy and Power Engineering, Changchun Institute of Technology, Changchun 130012, China; wangqingjia@stu.ccit.edu.cn; 2 State Key Laboratory of Power Systems, Department of Energy and Power Engineering, Tsinghua University, Beijing 100084, China; wuhw22@mails.tsinghua.edu.cn (H.W.); zhangman@mail.tsinghua.edu.cn (M.Z.); qsong@mail.tsinghua.edu.cn (Q.S.)

**Keywords:** urea, cyanuric acid, ammonia, pyrolysis, Aspen Plus

## Abstract

In response to the issues of poor economic efficiency and high CO_2_ emissions in the urea-to-ammonia technology of thermal power plants, this paper innovatively proposes a new ammonia production process for thermal power plants. This process utilizes the waste heat of thermal power plant boilers and conducts urea pyrolysis through two-stage heating to prepare ammonia and cyanuric acid. From this, the prepared ammonia can be used in the denitrification process of thermal power plants, and the prepared cyanuric acid can bring additional benefits to thermal power plants. The optimal process scheme was determined through orthogonal experiments of urea pyrolysis. And with the help of Aspen Plus software, a whole-process modeling analysis of urea pyrolysis experiments was carried out to investigate the influences of the melting temperature, melting time, reaction temperature, and reaction time on the process. The results show that when the melting temperature was 160 °C, the melting time was 45 min, the reaction temperature was 240 °C, and the reaction time is 20 min, which was the best scheme, 18.45% ammonia and 52.35% cyanuric acid could be obtained. Through the combined analysis of the Aspen Plus simulation and urea pyrolysis experiments, it was found that the melting temperature should be controlled within 160–167 °C, the melting time should be controlled within 40–45 min, the reaction temperature should be controlled within 240–245 °C, and the reaction time should be controlled within 15–20 min. Compared with the existing urea-to-ammonia process, this process has the advantages of nearly zero emissions and good economic benefits, thus providing reliable research data support for future industrialization.

## 1. Introduction

In the process of flue gas denitrification in thermal power plants, a large amount of ammonia is required as a reducing agent [1]. At present, there are mainly two stable sources of ammonia in thermal power plants. One is liquid nitrogen. However, liquid ammonia has characteristics such as being flammable, explosive, toxic, and corrosive, which are major safety hazards. There are serious safety risks in its transportation and large-scale storage [2,3]. The other source of ammonia is urea. Due to the characteristics of urea being non-toxic and easy to transport and store, it has gradually become a safe and reliable source of ammonia for thermal power plants [4,5,6]. But in the current process of urea pyrolysis for ammonia production, about 50% of the mass fraction of carbon and oxygen elements in urea is emitted in the form of CO_2_ [7,8], which is not only harmful to the environment but also increases operating costs.

Yao et al. [9] found that the decomposition of urea at lower temperatures is accompanied by a series of complex intermediate reactions while pyrolyzing urea to produce ammonia. These reactions lead to the transformation of the carbon and oxygen in urea into derivatives like cyanuric acid, thereby achieving near-zero carbon emissions [10]. Cyanuric acid is a downstream product of urea, which is widely used in industrial production, including construction, textile, rubber, and other industries, as well as in agriculture and medicine [11]. Cyanuric acid has a low toxicity, is a bactericidal and bleaching agent, and has a high market price [12,13,14]. The main production methods of cyanuric acid at home and abroad are urea pyrolysis methods. This pyrolysis method is mainly divided into two types: the solid-phase methods and liquid-phase methods. The solid-phase methods mainly include the direct pyrolysis of urea and molten salt pyrolysis. However, it generally has problems such as a low yield, harsh operating conditions, serious pollution, and low product purity [15,16]. Zhang et al. [17] used o-nitrotoluene as a solvent to synthesize cyanuric acid by a liquid-phase method, with a yield as high as 85%. However, o-nitrotoluene solvent is highly toxic and not suitable for the industrial preparation of cyanuric acid. Yan [18] and Liu et al. [19] used nitrobenzene as a solvent, and the yield of cyanuric acid reached 75%. Nitrobenzene is highly toxic and expensive, and the treatment of the three wastes generated in the production process is difficult. Therefore, it is also not suitable for large-scale industrial production. Zhang et al. [20] prepared cyanuric acid by cracking urea in silicone oil. The output of cyanuric acid can reach 93%, and silicone oil can be reused with little loss. However, this method is limited by problems such as the high viscosity of silicone oil, a complicated post-treatment, and a yellowish product color.

Liu et al. [21] proposed a scheme for the co-production of ammonia-cyanuric acid by the microwave pyrolysis of urea because of the disadvantages of the high risk of the liquid ammonia method and the high operating cost of the urea method in the current ammonia production process for denitration in thermal power plants. The yield of cyanuric acid can reach 70% when the mass ratio of urea to ammonium chloride is 36:1. This research result provides new inspiration for the preparation of denitration agents from urea in thermal power plants, and at the same time, the potential possibility of the co-production of cyanuric acid has attracted attention. Wang et al. [22] further improved the microwave pyrolysis cogeneration of ammonia-cyanuric acid process on the basis of Liu and compared it with the current urea pyrolysis ammonia production process in thermal power plants. In the preparation of cyanuric acid, an overall ammonia yield of 15.8% can be achieved. However, the microwave pyrolysis technology cannot achieve continuous production [23,24] and is difficult to scale up to an industrial scale.

Many scholars researched the reaction kinetics of urea pyrolysis. Ebrahimian et al. [25] established a droplet evaporation model of urea aqueous solution and constructed a 12-step reaction kinetics model. This model considers the generation of ammonia and isocyanic acid, and the generation and decomposition of biuret, cyanuric acid, and homologs. The movement inside the reactor was simulated with the help of CFD to intuitively understand the concentration distribution of decomposition products. Brack et al. [26] obtained the chemical reaction rates of the mutual transformation of four states through multiple thermogravimetric analyses combined with numerical simulation software expansion. This model consists of 15 elementary reactions, including the formation process of each intermediate product and the chemical kinetic parameters of various states. Birkhold et al. [27] calculated the activation energy and pre-exponential during the pyrolysis and hydrolysis of urea based on the experimental data of urea injection by combining fast-mixing and diffusion models using the least squares method, which was in agreement with the results of the data of Kim et al. [28]. Also, the experimental data were used to derive a pattern of the variation in the total reaction rate constant with temperature. The results obtained under the transformation of the Arrhenius diagram at the corresponding temperature were analyzed and verified by Birkhold et al. [29]. However, the recent related research mainly focused on the reaction kinetics of cyanuric acid preparations and has not yet involved the research on the co-production of ammonia-cyanuric acid in thermal power plants.

This paper innovatively proposes a process for the co-production of ammonia and cyanuric acid through urea pyrolysis. The main goal was to produce the ammonia required by thermal power plants, and the co-production of cyanuric acid brings additional benefits. This paper addresses the importance of ammonia in denitrification in thermal power plants and the problems of existing ammonia sources, thus leading to the introduction of the innovative process. Then, the experimental methods, processes, and analysis and determination methods are elaborated in detail. Based on the thermal stability analysis, urea pyrolysis experiment analysis, ammonia release characteristic analysis, and Aspen Plus (v12) process analysis, the optimal experimental conditions and the influence of various factors are presented. Finally, compared with other scholars’ research, the innovation and significance of this study are highlighted, and the main conclusions, such as the thermal stability results of urea and its products, the optimal reaction conditions, and the control parameter ranges, are summarized.

## 2. Materials and Methods

### 2.1. Research Method

**Figure 1:**
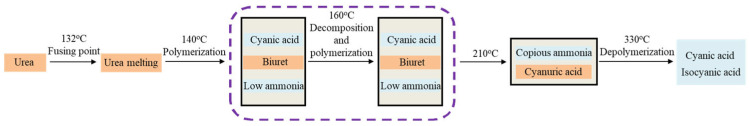
Urea pyrolysis flow chart.

This process aimed to produce the ammonia required by thermal power plants as the main goal and generate additional benefits through the co-production of cyanuric acid. In this process scheme, an innovative two-stage heating method of urea pyrolysis for preparing ammonia and cyanuric acid is proposed and it is divided into a preheating stage and a reaction stage. In the preheating section, urea undergoes condensation reactions to produce biuret and ammonia, and at the same time, reacts with isocyanic acid to generate biuret and ammonia. In addition, the further reaction of biuret and urea will produce biuret, triuret, and ammonia. With the increase in temperature, a large amount of ammonia is rapidly produced. It has a fast response speed and a short response time, which helps to solve the problem that the slow start-up time in the flue gas denitrification process leads to the inability to effectively remove nitrogen oxides in the flue gas. After entering the reaction section, the remaining materials will fully react to produce cyanuric acid. This solves the carbon emissions problem and brings additional benefits to the urea pyrolysis process. The reaction equations are detailed as shown in reactions (1) through (4). The flow chart is shown in Figure 1. Based on the urea pyrolysis experiment, a co-production process of ammonia-cyanuric acid that meets the ammonia demand of thermal power plants is proposed. Using Aspen Plus software, combined with the kinetic parameters of different reactions and using the waste heat of the boiler flue gas of thermal power plants as an external heat source, a full-process modeling analysis of the co-production process of urea pyrolysis was carried out. In the analysis of the simulation experiment results, statistical analysis was implemented to determine the significant differences between the experimental data and the simulation results. In addition, the influences of melting temperature, melting time, reaction temperature, and reaction time on the process parameters are also discussed.

2NH_2_CONH_2_ → NH_2_CONH_2_CONH_2_ + NH_3_ (1)

NH_2_CONH_2_ + HNCO → NH_2_CONH_2_CONH (2)

NH_2_CONHCONH_2_ + NH_2_CONH_2_ → NH_2_CONHCONHCONH_2_ + NH_3_ (3)

NH_2_CONHCONHCONH_2_ → C_3_H_3_N_3_O_3_ + NH_3_ (4)

### 2.2. Experimental Instruments and Raw Materials

The experimental instruments and raw materials employed in this particular study consisted of a thermogravimetric analyzer (NETZSCH TG209F3, NETZSCH, Dusseldorf, Germany), which is known for its high precision and reliability in analyzing thermal decomposition processes. Additionally, there was a constant temperature oil bath pot (HH-4T, Dingxinyi Experimental Equipment Co., Ltd., Shenzhen, China), which provided a stable and controlled heating environment. A thermocouple (WRNK-191, Mack Instrument, Huaian City, China) was used to measure the temperature accurately. A pH meter (DELTA 320, METTLER TOLEDO, Zurich, Switzerland) was utilized for determining the acidity or alkalinity of solutions. A pressure gauge (Y-100, Kaoyuan Instrument and Meter Co., Ltd., Shanghai, China) helped in monitoring pressure changes. As for the raw materials, industrial-grade urea (Xinghengtai, Wuhan, China) was used, along with boric acid (AR, Nanjing Reagent, Nanjing, China), concentrated hydrochloric acid (AR, Nanjing Reagent, Nanjing, China), silicone oil (AR, Nanjing Reagent, Nanjing, China), and methyl orange (AR, Nanjing Reagent, Nanjing, China).

### 2.3. Experimental Process

**Figure 2:**
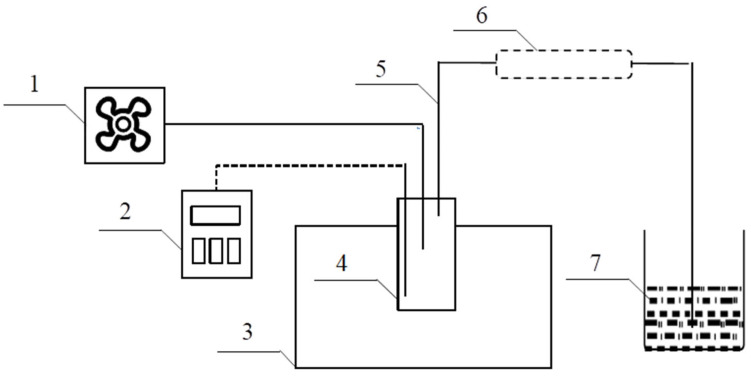
Experimental device. 1—gas-blowing device; 2—thermocouple; 3—oil bath; 4—reactor; 5—gas delivery line; 6—condenser; 7—ammonia collection device.

The urea pyrolysis device used in this study is shown in Figure 2. Before the experiment started, the oil bath was preheated to the melting temperature, and then the urea sample was put into the reactor. After the melting stage was completed, the temperature of the oil bath was raised to the reaction temperature. During the heating process, the urea continuously generated cyanuric acid and ammonia. In order to enable ammonia to be discharged from the reactor in time, a blowing device was added. During the process of heating the urea, some urea volatilized due to heat and was discharged in the form of gas, together with ammonia. In order to ensure the accuracy of the experimental results, a condenser was added. When the volatile urea gas passed through the condenser, it adhered to the condenser wall in the form of crystals. After the ammonia passed through the condenser, it was absorbed by the boric acid solution. The boric acid solution that absorbed ammonia was titrated with hydrochloric acid every ten minutes to determine the ammonia production. After the reaction was completed, the reactor was taken out of the oil bath and purging continued for 5 min to prevent residual ammonia from affecting the accuracy of the data. The reactor was left to cool to room temperature, and then the production of cyanuric acid in the reactor was measured.

### 2.4. Analytical Determination Method

In this study, the production of cyanuric acid was determined by high-performance liquid chromatography (HPLC). The production of ammonia was determined by hydrochloric acid titration. The titration test of the absorbed boric acid solution was carried out by using the prepared 1.208 mol/L hydrochloric acid solution. The consumption of hydrochloric acid *V* (milliliter) was recorded to calculate the production of ammonia. The reaction equations were as follows:

NH_3_ + HCI → NH_4_CI (5)

The amount of ammonia produced could be calculated from the reaction formula, which could be obtained by a calculation based on Formula (6):

(6) $$M=\frac{1.208\times V}{1000}\times \frac{V_{0}}{10}\times 17$$

In the formula:

- *M*: ammonia production, g;
- *V*: total volume of absorbed boric acid, mL;
- *V*_0_: volume of consumed hydrochloric acid, mL.

## 3. Results

### 3.1. Thermal Stability Analysis

**Figure 3:**
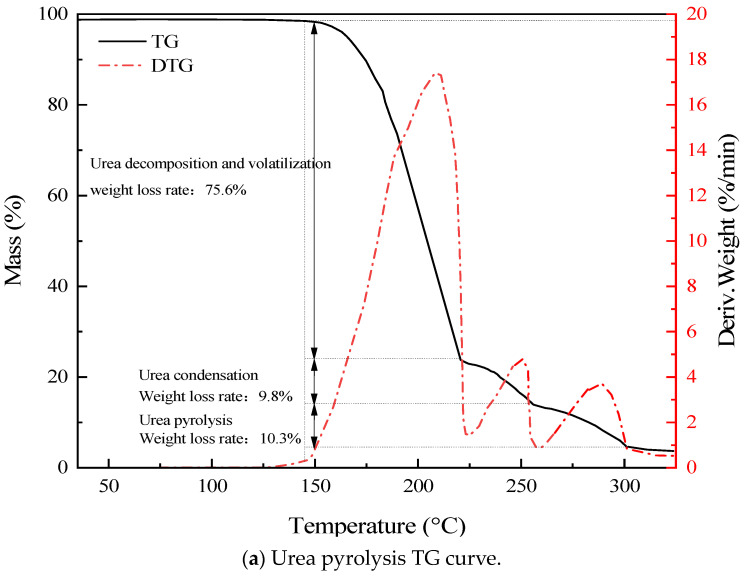

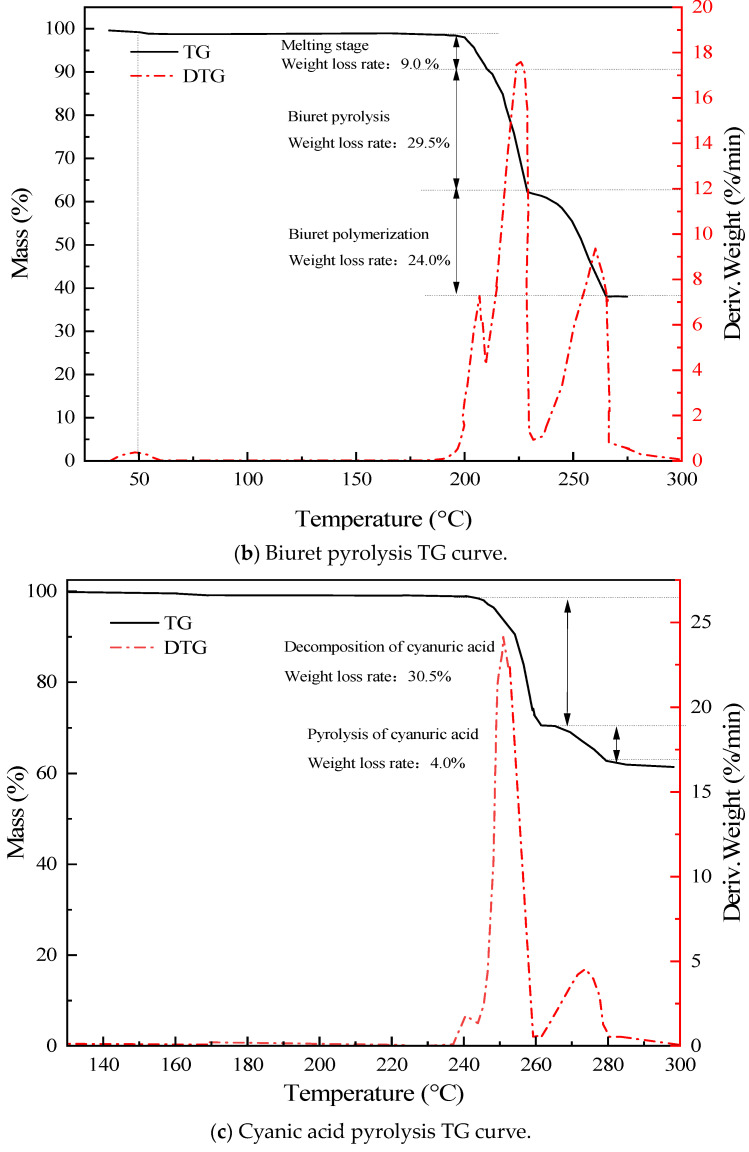
Materials TG curves.

The heating decomposition of urea is a multi-step reaction process that generates products such as ammonia, isocyanic acid, biuret, and cyanuric acid. To study the variation law of temperature during the urea pyrolysis process and thereby determine the temperature boundary conditions of the urea pyrolysis experiment, a thermogravimetric analysis of the thermal effects of urea and its urea products was carried out. As shown in Figure 3, the urea started to decompose at 150 °C, and the biuret started to decompose at 190 °C. To ensure that more biuret was produced in the melting stage and the biuret did not decompose, the melting temperature needed to be set between 150 °C and 190 °C. The generation temperature of the cyanuric acid was 210 °C and it started to decompose at 260 °C. Therefore, the reaction temperature was set between 210 °C and 260 °C.

### 3.2. Urea Pyrolysis Experimental Analysis

**Figure 4:**
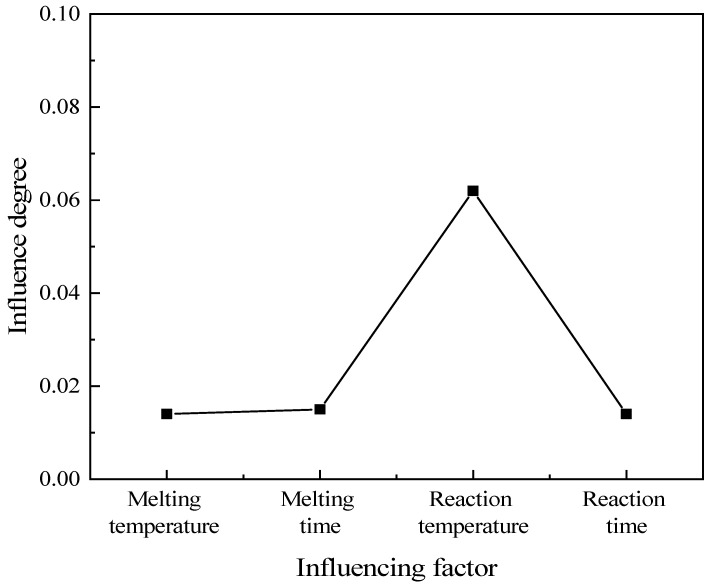
Analysis curve of extreme value results.

**Figure 5:**
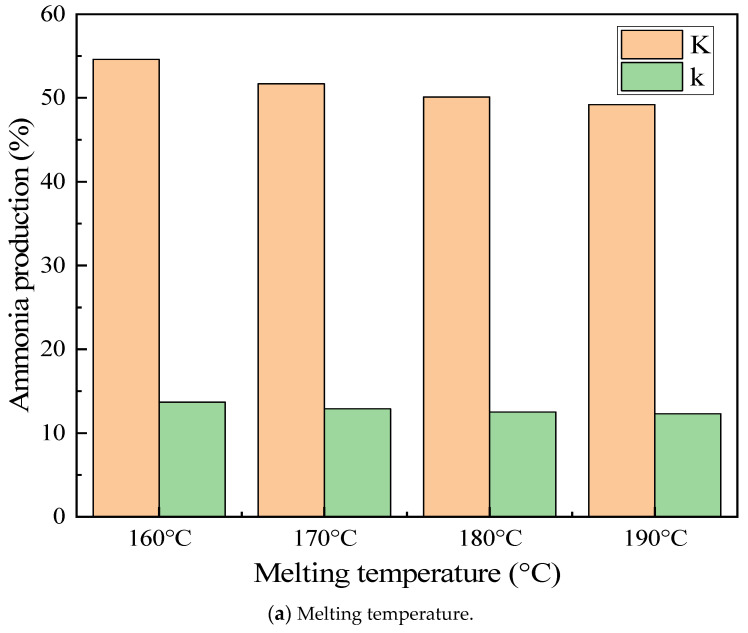

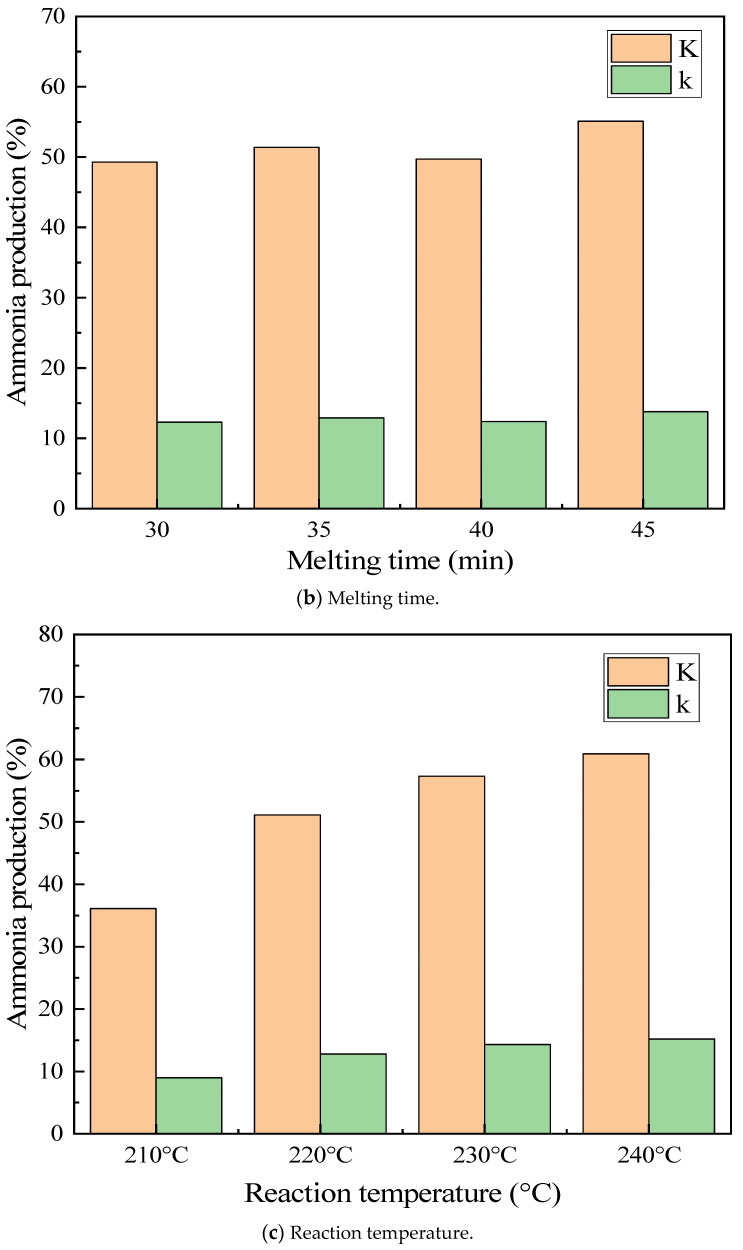

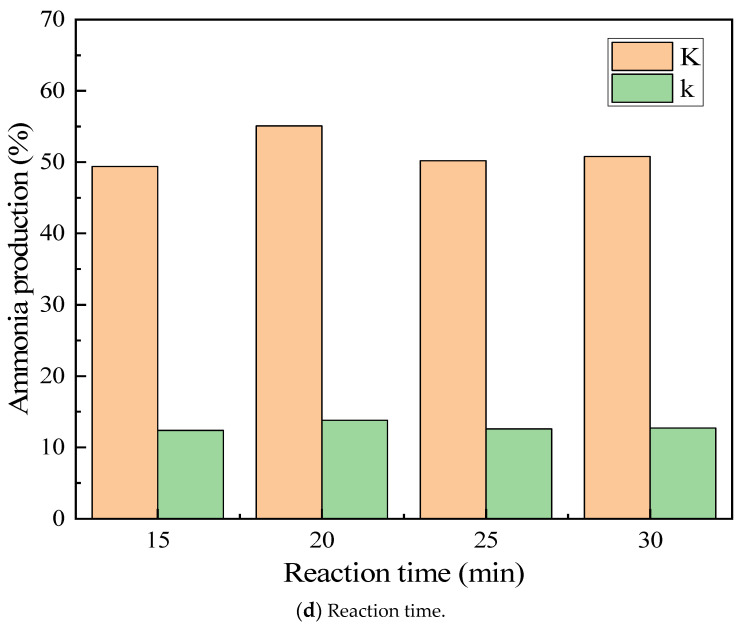
Level comparisons of the same factors. K: the sum of the experimental results for the same factors; k: the mean of the experimental results for the same factor.

The temperature boundary conditions of the preheater and the reactor were determined based on the thermogravimetric analysis experiment. An orthogonal table of four factors and four levels (L16(44)) was selected for the orthogonal experiment, and the experimental conditions were judged with ammonia production as the main index. The orthogonal experimental factors are shown in Table 1. The results of the orthogonal experiment are shown in Table 2. Different experimental conditions had obvious differences in ammonia production. The highest ammonia production could reach 17.4%, and the lowest was only 8.5%. To explore the influence degree of each influencing factor on ammonia production, the extreme value analysis was carried out as shown in Figure 4. Among the factors, the reaction temperature had the greatest influence on ammonia production, and the influence degrees of reaction time, melting temperature, and melting time on ammonia production were relatively close. To obtain the optimal preparation scheme, as shown in Figure 5, the ammonia production was compared at the same factor level. Within the variation range of each factor, the highest ammonia production occurred at the melting temperature of 160 °C, the melting time of 45 min, the reaction temperature of 240 °C, and the reaction time of 20 min. Therefore, the melting temperature of 160 °C, the melting time of 45 min, the reaction temperature of 240 °C, and the reaction time of 20 min formed the optimal scheme for this system.

To further verify the optimal scheme, experiments were continuously conducted. The release time and output of ammonia were recorded in real-time, and the output of cyanuric acid was determined. The verification experiment situation is shown in Table 3. Under the optimal scheme, the output of ammonia reached the maximum value of 18.5%, and the output of the prepared cyanuric acid was 52.53%. Under this experimental condition, high-output ammonia and relatively high-output cyanuric acid could be produced.

### 3.3. Ammonia Release Characteristics Analysis

**Figure 6:**
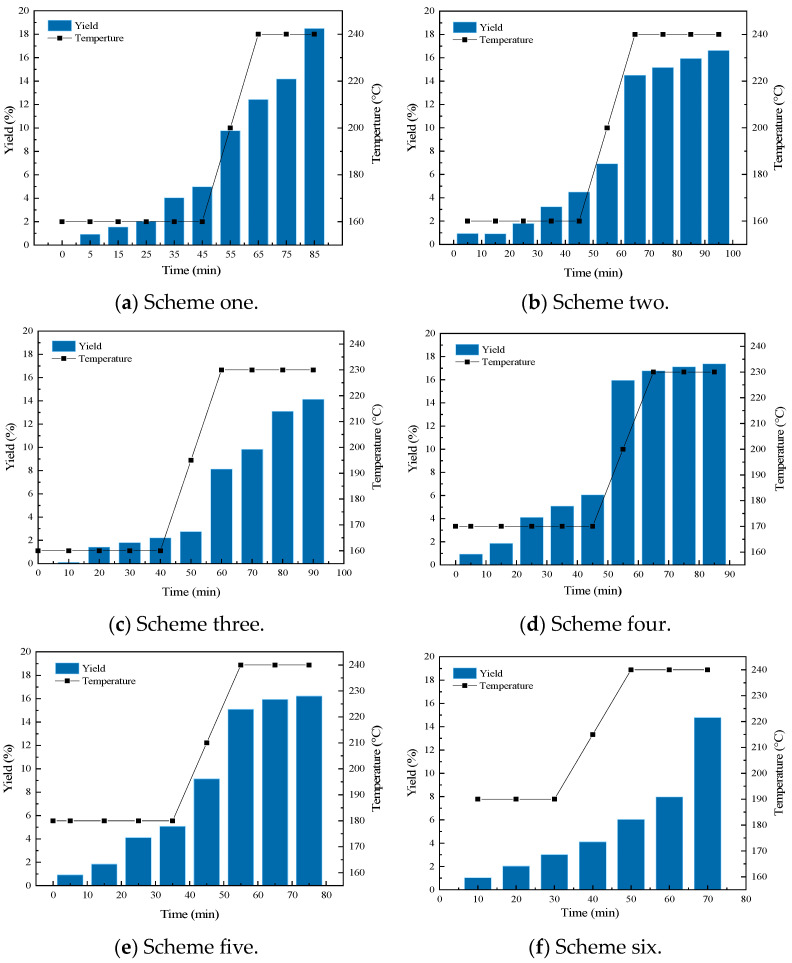
Ammonia production under each scheme.

Since the main aim of this process was to produce ammonia for denitrification in the boilers of thermal power plants, more focus was placed on the release characteristics of ammonia. To further analyze the release characteristics of ammonia, six groups of urea pyrolysis schemes that had the highest ammonia production in the experiments were selected. The data were analyzed to better understand the ammonia release characteristics and patterns during urea pyrolysis. The six groups of experimental schemes are shown in Table 4. The ammonia production under each reaction condition is shown in Figure 6. Ammonia began to be produced in the first ten minutes after the start of the experiment and continued until the end of the experiment. The process of ammonia generation could be divided into two stages: The first stage was the urea-preheating stage. In this stage, a small amount of ammonia was produced and the ammonia production was relatively stable. The second stage was the heating pyrolysis stage. With the increase in temperature, a large amount of ammonia was produced, with a fast response speed and a short response time. This helped to solve the problem that in the process of flue gas denitrification, the starting time was relatively slow and the nitrogen oxides in the flue gas could not be effectively removed. Overall, the production in the heating pyrolysis stage under various reaction conditions was higher than that in the preheating stage. With the increase in temperature, the ammonia production increased rapidly, the response speed was fast, and the response time was short.

### 3.4. Aspen Plus Process Analysis

#### 3.4.1. Aspen Plus Modeling

**Figure 7:**
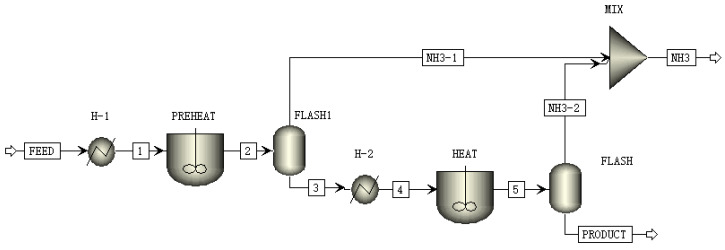
Flow chart of the co-production process of ammonia-cyanuric acid.

The urea pyrolysis process involves multiphase transformations. The kinetic parameters of each reaction affected the uniformity of the products and the accuracy of the conversion rate results. The whole-process modeling and analysis of the process of co-producing ammonia-cyanuric acid through urea pyrolysis in thermal power plants was conducted using Aspen Plus. The simulation was divided into two stages. The first stage was the preheating stage, and the second stage was the reaction stage. In this simulation, the external heating source was the heat exchange of the boiler flue gas in the thermal power plant. The Aspen Plus flow chart, unit module description, and reaction kinetic parameters of the ammonia-cyanuric acid co-production preparation system are presented in Figure 7 and Table 5 and Table 6.

#### 3.4.2. Analysis of Simulation Experiment Results

**Figure 8:**
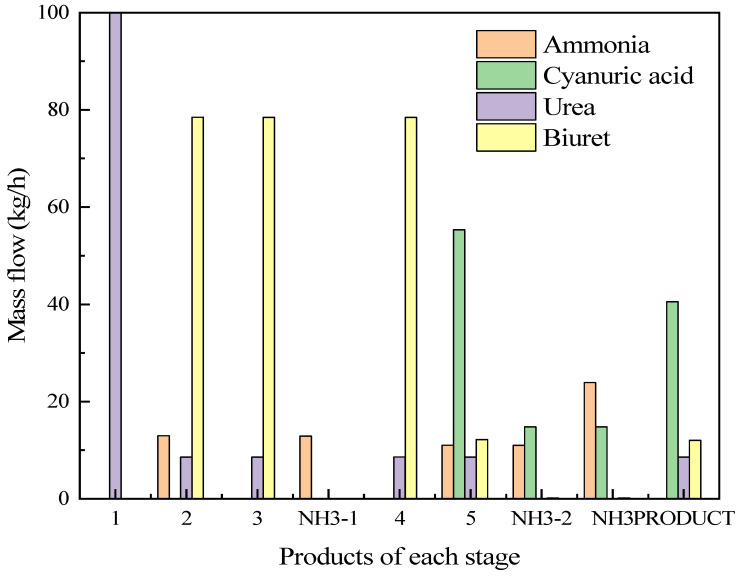
Simulation operation results.

**Figure 9:**
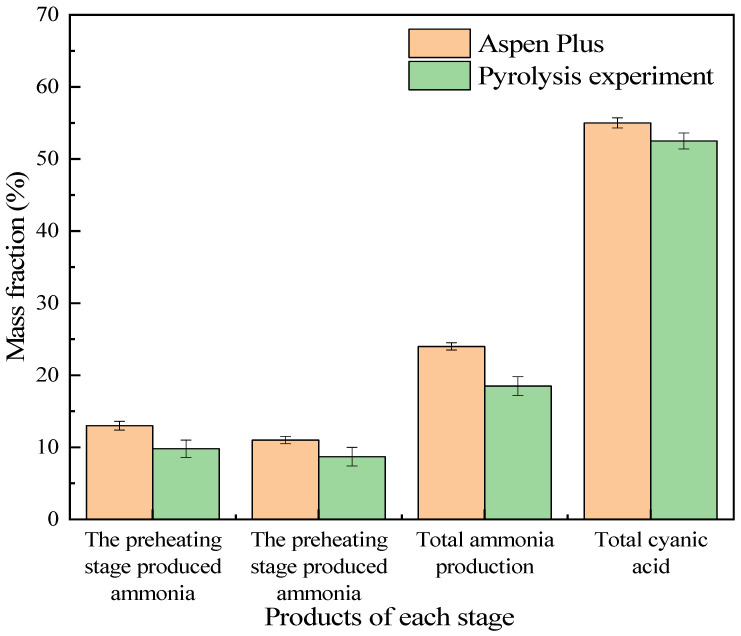
Comparison between simulation results and experimental results.

The Aspen Plus software was utilized to model the system for preparing ammonia and cyanuric acid through urea pyrolysis, simulate the production process of this system, and verify the accuracy of the urea pyrolysis experimental results and the feasibility of the process route. The simulation conditions adopted the optimal scheme of the urea pyrolysis experiment. The boundary condition settings of the ammonia-cyanuric acid co-production preparation model are shown in Table 7. The operation results are shown in Figure 8. Corresponding to the urea pyrolysis experiment, in the preheating stage, the main product was biuret and some ammonia was produced. With the increase in temperature, after the materials enter the reaction stage, a large amount of cyanuric acid and ammonia was produced. The comparison of production with the urea pyrolysis experiment is shown in Figure 9. According to the simulation results, urea produced 13% ammonia in the preheating stage and 11% ammonia in the reaction stage. The two stages produced a total of 24% ammonia and 55% cyanuric acid, and the entire pyrolysis process achieved energy and mass balances. It was found from the urea pyrolysis experiment that when the urea was pyrolyzed under optimal conditions, 9.8% ammonia could be produced in the preheating stage and 8.7% ammonia in the reaction stage. The two stages produced a total of 18.5% ammonia and 52.5% cyanuric acid. By comparing the simulation results with the experimental results, it was found that the amount of ammonia produced in each stage in the Aspen Plus simulation was greater than the experimental value. In the urea pyrolysis experiment, the thermal stability of urea itself was poor. At high temperatures, urea volatilizes and the degree of volatilization is intense, which results in the loss of some raw materials, and thereby directly reduces the production of ammonia and cyanuric acid. At the same time, the reaction conditions for side reactions during the urea pyrolysis process are special and the reactions are complex. In the current research, an accurate path for side reactions could not be accurately determined. Therefore, the occurrence of side reactions and the formation of by-products were not considered in the process simulation. It was assumed that the urea pyrolysis condensation reaction only produced two final products: cyanuric acid and ammonia. Therefore, the experimental result values were all lower than the process simulation result values.

#### 3.4.3. Melting Temperature Effect

**Figure 10:**
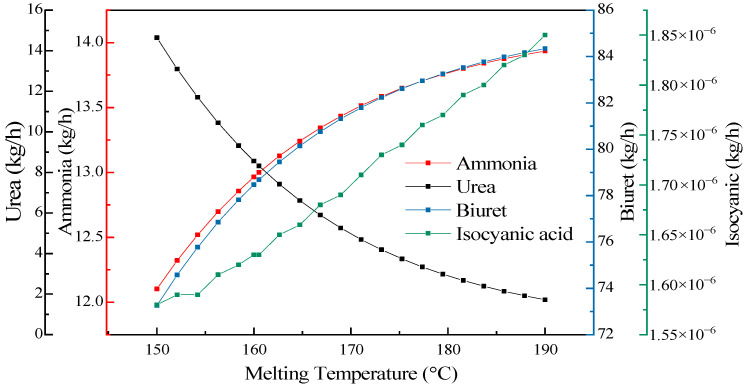
The influence of the melting temperature on the pyrolysis products.

To explore the influence of the melting temperature on the urea pyrolysis experiment while keeping other conditions unchanged, the set temperature of the preheating reactor was adjusted to change the melting temperature, and then the influence of the melting temperature on the mass flow distribution of products was analyzed. The influence of the melting temperature variation on the pyrolysis products of urea is shown in Figure 10. As the temperature continuously rose from 150 °C to 190 °C, the mass of each product increased with the increase in temperature. Urea generated ammonia and isocyanic acid through two steps of reactions and underwent a condensation reaction with isocyanic acid to form biuret. Isocyanic acid was both a product and a reactant. It can be seen from the trend of the curve in the figure that the mass of isocyanic acid showed an overall upward tendency, which indicates that the decomposition reaction of urea outweighed the condensation reaction of urea. Urea itself had poor thermal stability and began to decompose when the temperature reached 150 °C. As the temperature rose, the rate of the decomposition reaction gradually decelerated. When the temperature increased to 167 °C, the decomposition rate markedly slowed down, and the growth rate of ammonia production gradually slackened. Nevertheless, the urea pyrolysis experiment revealed that at 160 °C, there was a relatively high yield. Hence, when the melting temperature was controlled at between 160 °C and 167 °C, the ammonia production attained the maximum.

#### 3.4.4. Melting Time Effect

**Figure 11:**
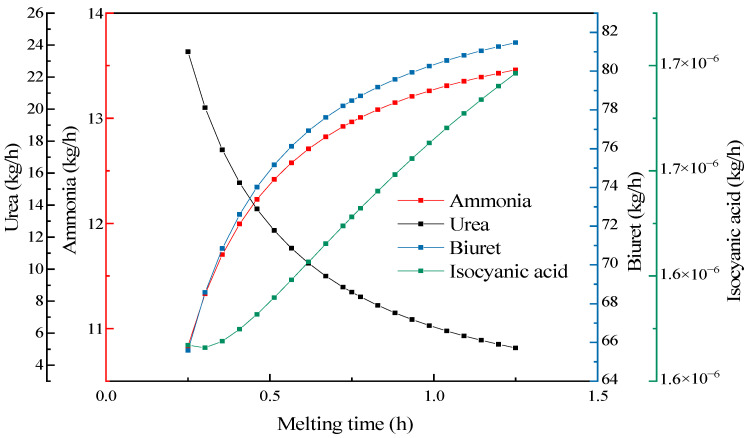
The influence of the melting time on the pyrolysis products.

The melting time (residence time) here denotes the period during which the substances remained in the reactor rather than the actual reaction time. To understand the influence degree of urea melting, while keeping other conditions unchanged and constantly changing the residence time of urea in the preheating system, the influence of the melting time on the mass flow distribution of products was analyzed. As shown in Figure 11, as the melting time increased, the contents of biuret, ammonia, and isocyanic acid significantly increased. When the melting time was longer, the reaction proceeded more sufficiently. However, a longer melting time was bound to cause an increase in the system energy consumption. When the melting time exceeded 40 min, the increased amplitude of the biuret was no longer obvious, while the urea pyrolysis experiment showed that a higher yield was achieved at 45 min. Therefore, the melting time should be controlled within the range of 40–45 min.

#### 3.4.5. Reaction Time Effect

**Figure 12:**
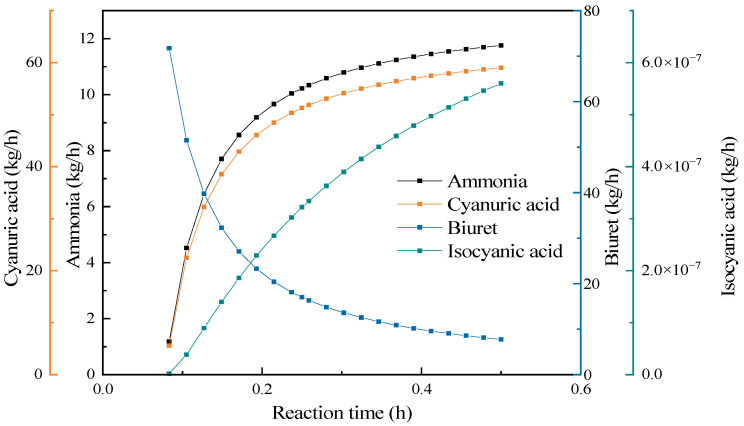
The influence of the reaction time on the pyrolysis products.

To understand the situation of the final products of urea and intermediate products with the change in residence time, while keeping other conditions unchanged, the residence time was changed to analyze the influence of the reaction time on the mass flow distribution of products, as shown in Figure 12. Urea was no longer the main reactant in the reaction stage. Biuret and isocyanic acid were the main reactants, which generated cyanuric acid, ammonia, and isocyanic acid through two types of condensation reactions. Among them, isocyanic acid acted as a reactant in the first type of condensation reaction and was a product in the second type of condensation reaction. With the progress of the reaction time, except for the decrease in the output of the biuret, the outputs of the other products significantly increased. It can be seen from the change curve of the isocyanic acid in the figure that while the isocyanic acid was reacting as a reactant, the output was still increasing, which indicates that the degree of the second type of condensation reaction was greater than that of the first type. The main reaction in the reaction stage was the second type of condensation reaction, namely, reaction R6 in Table 6 mentioned earlier. After 15 min, the growth rate of each product slowed down, while the urea pyrolysis experiment showed that a higher yield was achieved at 20 min. Therefore, the reaction time should be controlled within the range of 15–20 min.

#### 3.4.6. Reaction Temperature Effect

**Figure 13:**
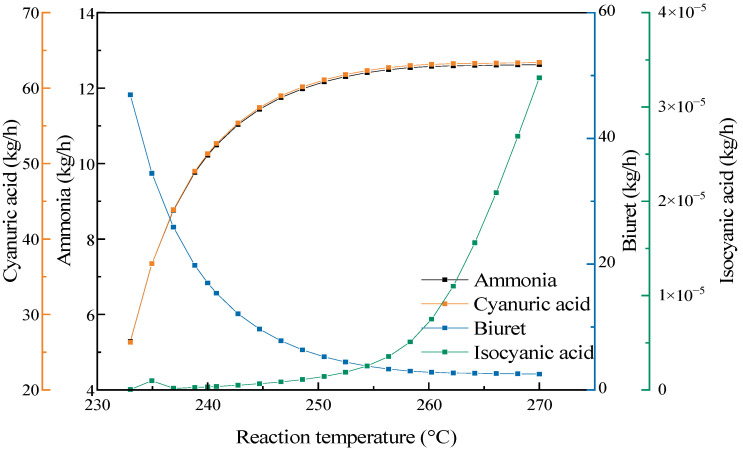
The influence of the reaction temperature on the pyrolysis products.

To explore the influence of the reaction temperature on the final products, while keeping other conditions unchanged, the reaction temperature was adjusted to analyze the impact of the reaction temperature on the mass flow distribution of the products. As shown in Figure 13, with the change in the reaction temperature, except for the decrease in the output of biuret, the outputs of the other products significantly increased. The outputs of ammonia and cyanuric acid increased as the reaction temperature rose, and the output of cyanuric acid was as high as 64.5 kg/h. When the temperature rose to 245 °C, the increase rate of the outputs of cyanuric acid and ammonia gradually slowed down. Through comparison with the change curves of the abovementioned influencing factors, it was found that changing the reaction temperature could greatly increase the outputs of ammonia and cyanuric acid. However, a higher reaction temperature could simultaneously cause the decomposition of cyanuric acid and the generation of complex by-products. The urea pyrolysis experiment indicated that a higher yield was achieved at 240 °C. Therefore, the reaction temperature should be controlled within the range of 240–245 °C.

## 4. Discussion

In the field of urea pyrolysis and the co-production of ammonia-cyanuric acid, some scholars conducted relevant research due to the shortcomings of high operating costs and large carbon emissions in the production of ammonia from urea pyrolysis [30,31,32], as well as the problems of low yield and poor purity in the solid-phase method [33,34] and high operating costs in the liquid-phase method [35,36] for the preparation of cyanuric acid by urea pyrolysis. Liu [37] and Zhu et al. [38,39] proposed a scheme for the co-production of ammonia-cyanuric acid by the microwave pyrolysis of urea, and the yield of cyanuric acid in this process can reach 70%. Wang et al. [22] further improved the microwave pyrolysis co-production process of ammonia-cyanuric acid and achieved a total ammonia yield of 15.8% in the preparation of cyanuric acid.

In our research, we innovatively proposed a process for the co-production of ammonia and cyanuric acid by urea pyrolysis through a two-stage heating method. The orthogonal experiment results showed that when the melting temperature was 160 °C, the melting time was 45 min, the reaction temperature was 240 °C, and the reaction time was 20 min, the ammonia yield was the highest, where 18.45% ammonia and 52.35% cyanuric acid could be produced by pyrolysis. Compared with previous studies, our process achieved relatively high ammonia yield and cyanuric acid production, and the reaction conditions were also optimized. In addition, our research also focused on the analysis of the thermal stability of urea and its products, as well as the release characteristics of ammonia. The thermogravimetric experiment showed that urea and biuret started to decompose at 150 °C and 190 °C, respectively, and the generation and decomposition temperatures of cyanuric acid were also determined. The analysis of the ammonia release characteristics showed that in the heating pyrolysis stage, the ammonia output increased rapidly, with a fast response speed and a short response time, which was beneficial for the effective removal of nitrogen oxides in the flue gas denitrification process.

The research results of this study have important implications for the thermal power industry. First, the co-production of ammonia and cyanuric acid by urea pyrolysis can make full use of the waste heat of the thermal power plant boiler, reduce the dependence on external energy sources, and achieve energy conservation and emission reduction. Second, the prepared ammonia can be used in the denitrification process of the thermal power plant, thus reducing the emission of nitrogen oxides and environmental pollution. At the same time, the prepared cyanuric acid can bring additional economic benefits to the thermal power plant and improve the economic efficiency of the enterprise.

## 5. Conclusions

This study innovatively proposed a new process for the co-production of ammonia-cyanuric acid by utilizing the waste heat of the boiler in thermal power plants and conducting urea pyrolysis through two-stage heating; furthermore, it determined the boundary conditions of the process through experiments and software analysis. This process was novel, as it optimized the reaction conditions and increased the yield of ammonia and cyanuric acid. The research results on the thermal stability of urea and its products and the release characteristics of ammonia can provide references for other related studies, and they are replicable. At the same time, this study provided a new ammonia production process for thermal power plants, which can save energy and reduce emissions, reduce nitrogen oxide emissions and environmental pollution, bring additional economic benefits, and provide reliable data support for industrial production, which makes a positive contribution to this research line.

The main research conclusions were as follows: The thermogravimetric experiment showed that urea and biuret started to decompose at 150 °C and 190 °C, respectively, with the generation temperature of cyanuric acid being 210 °C, and it decomposed at 260 °C. The orthogonal experiment analysis revealed that when the melting temperature of urea was 160 °C, the melting time was 45 min, the reaction temperature was 240 °C, and the reaction time was 20 min, the ammonia yield was the highest, where 18.45% ammonia and 52.35% cyanuric acid could be produced by pyrolysis. At the same time, under various reaction conditions of urea, the output in the heating pyrolysis stage was higher than that in the preheating stage. With the increase in temperature, the ammonia output increased rapidly, with a fast response speed and a short response time. The combined analysis of the Aspen Plus simulation and urea pyrolysis experiments indicated that when the melting temperature was controlled at 160–167 °C, the melting time should be controlled at 40–45 min; furthermore, the reaction time should be controlled at 15–20 min and the reaction temperature should be controlled at 240–245 °C so that the ammonia output can reach the maximum value.

## Figures and Tables

**Table 1:** Orthogonal experimental design of four factors and four levels.

	Factor	Melting Temperature (°C)	Melting Time (Minutes)	Reaction Temperature (°C)	Reaction Time (Minutes)
Level					
1		45.6	1.63 × 10−9	533	59.4
2		62.4	3.06 × 10−9	663	66.3
3		58.5	2.69 × 10−9	386	55.2
4		53.6	2.26 × 10−9	686	62.4
5		64.5	3.27 × 10−9	494	61.8
6		84.2	5.56 × 10−9	469	74.0

**Table 2:** Orthogonal experiment results.

ID	Melting Temperature (°C)	Melting Time (Minutes)	Reaction Temperature (°C)	Reaction Time (Minutes)	Ammonia Yield (%)
1	160	30	210	15	10.0
2	160	35	220	20	13.9
3	160	40	230	25	14.1
4	160	45	240	30	16.6
5	170	30	220	25	11.8
6	170	35	210	30	8.7
7	170	40	240	15	13.8
8	170	45	230	20	17.4
9	180	30	230	30	12.7
10	180	35	240	25	15.8
11	180	40	210	20	9.0
12	180	45	220	15	12.6
13	190	30	240	20	14.8
14	190	35	230	15	13.1
15	190	40	220	30	12.8
16	190	45	210	25	8.5

**Table 3:** Yield of ammonia and cyanic acid under the optimal scheme.

	Melting Temperature (°C)	Melting Time (Minutes)	Reaction Temperature (°C)	Reaction Time (Minutes)	Ammonia Production (%)	Cyanic Acid Production (%)
Optimal scheme	160	45	240	20	18.45	52.53

**Table 4:** Urea pyrolysis scheme.

Name	Melting Temperature (°C)	Melting Time (Minutes)	Reaction Temperature (°C)	Reaction Time (Minutes)	Ammonia Production (%)
Scheme one	160	45	240	20	18.5
Scheme two	160	45	240	30	16.6
Scheme three	160	45	230	25	14.1
Scheme four	170	45	230	20	17.4
Scheme five	180	35	240	20	16.8
Scheme six	190	30	240	20	14.8

**Table 5:** Each unit module of the simulation process of ammonia-cyanic acid co-production.

Name	Heating Module	Reaction Module	Separation Module	Heating Module	Reaction Module	Separation Module	Mixing Module
ID	H-1	PREHEAT	FLASH1	H-2	HEAT	FLASH	MIX

**Table 6:** Chemical kinetic parameters of urea pyrolysis reaction.

Number	Chemical Reaction Equation	Pre-Exponential Factor	Activation Energy (kJ/mol)
R1	CO(NH_2_)_2_ → NH_3_ + HNCO	3 × 10^4^	84.42
R2	CO(NH_2_)_2_ + HNCO → C_2_H_5_N_3_O_2_	3.3 × 10^10^	85.54
R3	C_2_H_5_N_3_O_2_ → CO(NH_2_)_2_ + HNCO	5.626 × 10^24^	266.38
R4	CO(NH_2_)_2_ → NH_3_ + HNCO	3 × 10^4^	84.42
R5	C_2_H_5_N_3_O_2_ + HNCO → C_3_H_3_N_3_O_3_ + NH_3_	2.5031 × 10^19^	153.12
R6	2C_2_H_5_N_3_O_2_ → C_3_H_3_N_3_O_3_ + HNCO + 2NH_3_	2.5031 × 10^19^	254.12

**Table 7:** System boundary conditions for the co-production of ammonia and cyanuric acid.

Module	Name	Parameter	Unit	Value
Urea	FEED	Flow rate	kg/h	100
Urea	FEED	Temperature	°C	25
Heating module	H-1	Temperature	°C	160
Reaction module	PREHEAT	Temperature	°C	160
Reaction module	PREHEAT	Residence time	min	45
Separation module	FLASH1	Temperature	°C	160
Heating module	H-2	Temperature	°C	240
Reaction module	HEAT	Temperature	°C	240
Reaction module	HEAT	Residence time	min	20
Separation module	FLASH	Temperature	°C	240
Mixing module	MIX	Pressure	kPa	101.325

Note: The pressure setting in all pyrolysis modules was normal pressure, and thus, it is not listed in detail in the table.

## Data Availability

The original contributions presented in the study are included in the article, further inquiries can be directed to the corresponding authors.
